# Improved YOLOv5 Network for High-Precision Three-Dimensional Positioning and Attitude Measurement of Container Spreaders in Automated Quayside Cranes

**DOI:** 10.3390/s24175476

**Published:** 2024-08-23

**Authors:** Yujie Zhang, Yangchen Song, Luocheng Zheng, Octavian Postolache, Chao Mi, Yang Shen

**Affiliations:** 1 Logistics Engineering College, Shanghai Maritime University, Shanghai 201306, China; 202130210133@stu.shmtu.edu.cn (Y.Z.); 202230210126@stu.shmtu.edu.cn (Y.S.); 202330210038@stu.shmtu.edu.cn (L.Z.); chaomi@shmtu.edu.cn (C.M.); 2 School of Technology and Architecture, ISCTE-Instituto Universitário de Lisboa, 1649-026 Lisbon, Portugal; 3 Instituto de Telecomunicações, ISCTE-Instituto Universitário de Lisboa, 1649-026 Lisbon, Portugal; opostolache@lx.it.pt; 4 Shanghai SMUVision Smart Technology Ltd., Shanghai 201306, China; 5 Higher Technology College, Shanghai Maritime University, Shanghai 201306, China

**Keywords:** container spreader, YOLOv5, machine vision, optical method, segmentation

## Abstract

For automated quayside container cranes, accurate measurement of the three-dimensional positioning and attitude of the container spreader is crucial for the safe and efficient transfer of containers. This paper proposes a high-precision measurement method for the spreader’s three-dimensional position and rotational angles based on a single vertically mounted fixed-focus visual camera. Firstly, an image preprocessing method is proposed for complex port environments. The improved YOLOv5 network, enhanced with an attention mechanism, increases the detection accuracy of the spreader’s keypoints and the container lock holes. Combined with image morphological processing methods, the three-dimensional position and rotational angle changes of the spreader are measured. Compared to traditional detection methods, the single-camera-based method for three-dimensional positioning and attitude measurement of the spreader employed in this paper achieves higher detection accuracy for spreader keypoints and lock holes in experiments and improves the operational speed of single operations in actual tests, making it a feasible measurement approach.

## 1. Introduction

**Figure 1:**
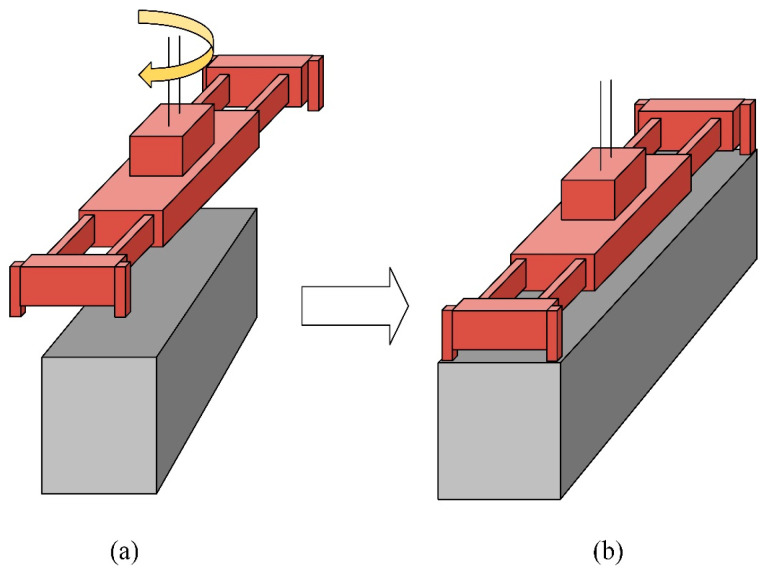
(**a**) Depicts the displacement and rotation between the spreader and the container; (**b**) depicts the aligned state of the spreader with the container.

In the operation of automated quayside container cranes, the three-dimensional position and rotational angles of the spreader are crucial parameters. Automated quayside container cranes are specifically designed for container terminals and are responsible for transferring containers between container trucks and container ships; a process known as container lifting operations. During the automated container lifting process, the spreader is first moved to an approximate position over the target container, then the spreader’s position is fine-tuned, and finally, the twist lock on the spreader relates to the container lock holes. As shown in Figure 1, Figure 1a depicts the displacement and rotation between the spreader and the container, and Figure 1b depicts the aligned state of the spreader with the container. This alignment work relies on the perception system’s accurate measurement of the spreader’s three-dimensional position and rotational angles, where the timeliness and accuracy of measurements are important factors affecting operational efficiency.

Currently, various sensor-assisted spreader positioning methods are used in engineering applications, primarily employing LiDAR (Light Detection and Ranging) to collect posture data of container spreaders for positioning. The advantage of LiDAR is its ability to support all-weather operations, which performs well in the unstable lighting conditions of container terminal environments [1,2]. However, disadvantages include susceptibility to interference in rainy and foggy weather conditions. Additionally, LiDAR presents challenges with complex installation and limited measurement range [3]. For example, in the positioning tasks of gantry cranes, the optimal installation location for LiDAR is on the crane’s crossbeam. However, due to the significant distance from the crossbeam to the containers on the ground, laser devices capable of precise positioning at such distances are expensive. If the radar is mounted on the legs of the gantry crane, the narrow field of view may result in measurement blind spots. These issues introduce certain limitations to the LiDAR approach in practical applications.

Thus, improving the accuracy and reliability of spreader position and orientation measurements remains an important research direction. Current proposals include vision-based object pose measurement systems, which exhibit good measurement accuracy in stable environments but still face challenges in complex measurement environments and with the limited computational resources in engineering applications.

This paper describes a method for detecting the three-dimensional position and rotational angles of a spreader using only a single visual camera. This method utilizes a pure visual detection approach without LiDAR, enhances the YOLOv5 network with an attention module, and integrates image morphological algorithms to reduce computational power consumption and increase the detection accuracy of keypoints on the spreader and lock holes in images, offering a feasible measurement for the spreader’s three-dimensional position and rotational angles.

The contributions of this paper are mainly reflected in the following aspects:

1. Addressing the limitations in LiDAR installation locations and lack of computational resources in engineering applications, a pure visual detection system using only a single camera has been proposed;
2. Considering the complex lighting conditions and noise issues of camera image samples in measurement environments, a pre-processing method for image samples has been proposed;
3. To overcome the limitations of the conventional YOLOv5 network in keypoint detection and small target detection, an attention module has been added to the network, enhancing the detection accuracy of keypoints on the spreader and the container lock holes and ultimately improving the measurement accuracy of the spreader’s three-dimensional position and rotational angles.

## 2. Related Works

Detection methods based on visual cameras are severely affected by strong sunlight or reliance on artificial light sources at night, leading to distortion of the original color and texture information in the images, which increases the difficulty of feature extraction and recognition for image processing algorithms. Additionally, in port environments, fine particles such as dust scatter light, reducing scene visibility and causing noise and blurring in images, which in turn leads to the loss of crucial detail features. To address the disturbances caused by non-uniform lighting conditions and other environmental factors, several common techniques for image enhancement are currently used: histogram equalization [4], Retinex theory [5], and methods using deep learning for image enhancement [6]. These techniques effectively improve image display under uneven lighting conditions. However, the limited computational power of hardware installed within the confined spaces of quayside cranes makes the use of complex deep learning methods for image enhancement a computational burden.

The core of histogram equalization algorithms is to enhance contrast by expanding the overall dynamic range of the image. Traditional histogram equalization methods often excessively enhance contrast, resulting in unnatural-looking images prone to visual distortions, and may amplify noise during the detail enhancement process. To address the issues of detail loss and increased noise that can arise from global histogram equalization, Contrast Limited Adaptive Histogram Equalization (CLAHE) has been proposed [7]. CLAHE has achieved some success in improving noise robustness, but there is still room for further improvement in enhancing local details and color naturalness. Additionally, Celik et al. [8] proposed a Context and Variance Contrast (CVC) enhancement algorithm that achieves non-linear pixel value mapping by analyzing contextual relationships between image pixels and their histograms, thereby enhancing low-light images. Although these methods have shown improvements in certain aspects, they typically perform modestly in noise reduction, especially for images with specific color distributions, and may even increase noise in some cases.

Methods based on Retinex theory [5], which hypothesize that an image can be decomposed into reflection and illumination components, are commonly used to adjust image brightness distribution to remove overexposure and enhance dark area details. The main challenge with Retinex methods is the selection of parameters. Most existing Retinex-based methods rely on the classic Multi-Scale Retinex (MSRCR) method [9] and carefully designed manual constraints and prior parameters for this highly uncertain decomposition [10,11]. However, the design of prior parameters may be limited by the parameter model when applied to different scenes. Chen Wei et al. [12] combined deep learning technology to propose Retinex-Net, which can be trained on given datasets. Its self-learning capability allows it to adapt to different scenes, achieving good low-light image enhancement and denoising effects. Similar to light enhancement algorithms, most existing image denoising methods still rely on prior parameters to adjust dehazing effects [13,14], achieving good results to some extent. However, due to the complex and variable nature of outdoor scenes, their application effects are not ideal because the adjustment of prior parameters can only be effective in certain applicable scenarios.

With the application of deep learning, recent years have seen the emergence of new methods using autonomously learning adaptive network structures [15,16] to automatically adjust and fit dehazing parameter designs corresponding to different images, effectively enhancing the robustness and generalization ability of dehazing algorithms. However, these methods lack sufficient empirical cases to demonstrate their final application effects.

In terms of object pose detection using visual cameras, the commonly used method is the PNP (Perspective-n-Point) algorithm, which performs well with fixed camera angles [17]. These pose detection methods rely on the detection of pixel coordinates of keypoints in the image. Yin Y. et al. [18] used YOLOv4 and YOLOv5s network models, incorporating improvements to the loss function at the center points of the bounding boxes, successfully solving the problem of keypoint detection and pose estimation when detected targets occlude each other. Lou H. et al. [19] proposed a small object detection algorithm based on YOLOv8 using depth-wise separable convolution, down sampling operations to extract feature information and improving the original model’s C2f module to achieve the fusion of different size features, thus enhancing the performance of the small object detection algorithm. However, despite improvements, the detection accuracy after lightweight processing still has significant room for improvement, and the misjudgment probability remains high in practical applications using keypoints for pose estimation. Zhang Qiang et al. [20] used attention mechanisms to locate target heatmaps, employing a mask cross-attention mechanism to optimize coarse-scale features and introducing fine-scale features to improve contour details, thereby enhancing the accuracy of target detection. Mi et al. [21] improved the detection accuracy of target poses by detecting standard parts with fixed sizes. Wang Juan et al. [22] proposed a multi-scale target detection algorithm based on the YOLO framework, combining a super-resolution reconstruction module and channel attention mechanism, effectively improving the detection accuracy of targets with large scale spans. Zwolfer M. et al. [23] studied the extraction of 2D keypoints and analyzed the performance of pose detection algorithms using 2D keypoints.

In summary, current image preprocessing methods have certain limitations in different environments and still require design for actual application scenarios. In the use of pure visual image pose detection methods, YOLO algorithms have shown good experimental results, but there is still significant room for improvement in the detection accuracy of keypoints and small targets, especially in specific port environments, where issues in measuring the three-dimensional position and rotational angle of spreaders still lack effective and reliable solutions.

## 3. Three-Dimensional Positioning and Attitude Measurement System

### 3.1. Hardware System

**Figure 2:**
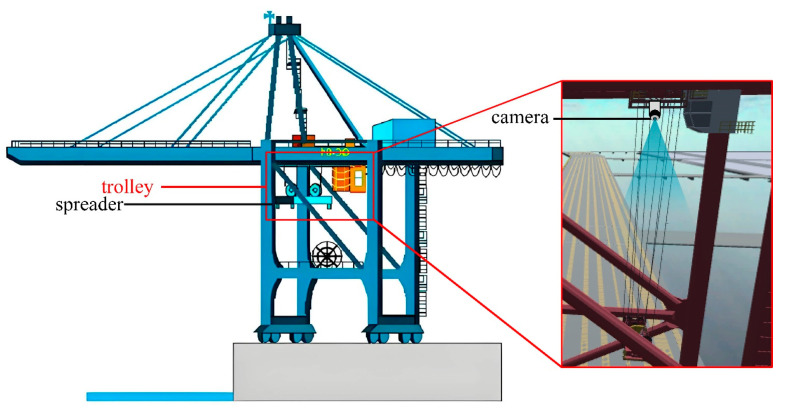
Equipment installation diagram.

This paper presents a hardware system for the three-dimensional positioning and attitude measurement of the spreader based on visual measurement, consisting of a single visual camera and a single-edge computing module. The visual camera in the system is a vertically mounted fixed-focus camera, affixed to the trolley frame of the quayside crane, as shown in Figure 2. The trolley is a mobile platform mounted on the boom of the quayside crane, capable of smooth operation along fixed tracks, driving the movement of the spreader during lifting operations. The spreader is connected to the trolley by steel cables, and as the trolley moves, the spreader will swing to some extent. The single-edge computing module is responsible for receiving and processing the images of the upper surface of the spreader collected by the visual camera. In practice, the visual camera continuously captures images of the spreader and transmits them to the single-edge computing module. The single-edge computing module analyzes these images through advanced image processing algorithms to accurately determine the position and attitude of the spreader.

**Figure 3:**
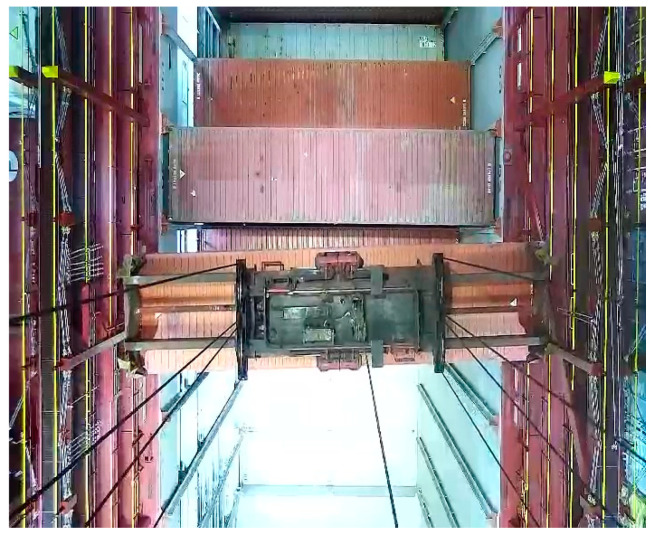
An image of the spreader captured by the camera.

During container loading operations, the spreader may experience changes in attitude such as twisting and shifting. The image of the spreader captured by the camera is shown in Figure 3, which depicts the spreader in a twisted position. Adjustments to the spreader’s twisting and shifting are made through the forward and backward movement of the trolley. The distance the trolley moves depends on the rotational angle and the offset of the spreader.

### 3.2. Algorithm Design

**Figure 4:**
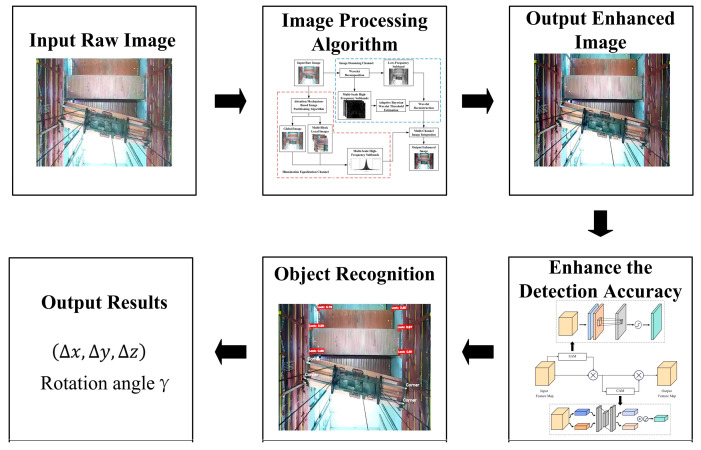
Algorithm design flowchart.

The workflow of the three-dimensional positioning and attitude measurement algorithm for spreaders based on visual measurement proposed in this paper is illustrated in Figure 4. Initially, a raw image is input; it then undergoes image preprocessing where a multi-channel image processing algorithm proposed in this paper is applied. This algorithm effectively balances the image’s lighting levels and reduces noise. After preprocessing, an enhanced image is output. For the enhanced image, keypoint and lock hole detection is necessary. To improve the detection accuracy of keypoints and small targets, an improved YOLOv5 algorithm is used, which includes an added attention module. Finally, by analyzing the detected image keypoints and container lock holes, the spreader’s rotation angle and offset distance relative to the baseline position are determined.

#### 3.2.1. Multi-Channel Image Processing Algorithm for Spreader Images

**Figure 5:**
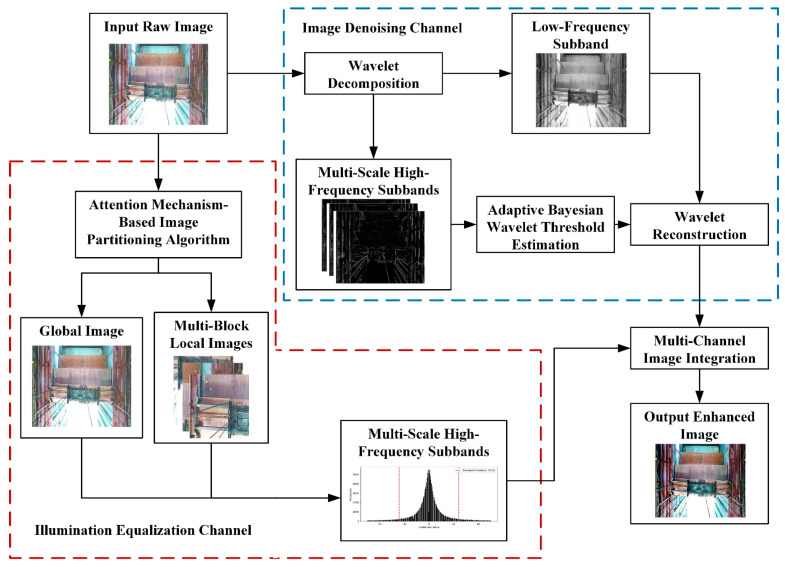
Flowchart of multi-channel image processing algorithm.

To address the interference problems caused by uncertain environmental conditions, this paper designs a multi-channel image enhancement algorithm that combines global and local scales for the spreader images at container terminal quaysides. This algorithm serves as a preprocessing part of the image detection algorithm to mitigate the impacts of lighting and visibility. As shown in Figure 5, the image processing workflow is divided into two parts: an image denoising channel and a lighting equalization channel.

**Lighting Equalization Channel.** In all-weather outdoor environments, images captured by cameras are subject to interference from sunlight and artificial lighting, and the uneven distribution of light can easily create overly bright or dark areas in images. This not only obscures key information in the images but may also prevent image recognition algorithms from accurately extracting the needed features, thereby affecting the judgment and decision-making of the entire automation system. To address these issues, this paper introduces a lighting equalization algorithm at the initial stage of the spreader image preprocessing workflow. This algorithm effectively adjusts the brightness distribution in images, ensuring that details under shadows or strong light exposure are clearly captured.

The lighting equalization channel designed in this paper, considering the computational burden that neural networks might introduce, employs an image partitioning method based on attention mechanisms and Retinex theory. According to different levels of environmental light reflection, the image is divided into multiple focused areas. A multi-stage Retinex algorithm is then used to adaptively enhance details in dark areas while simultaneously suppressing halo effects in bright areas.

**Figure 6:**
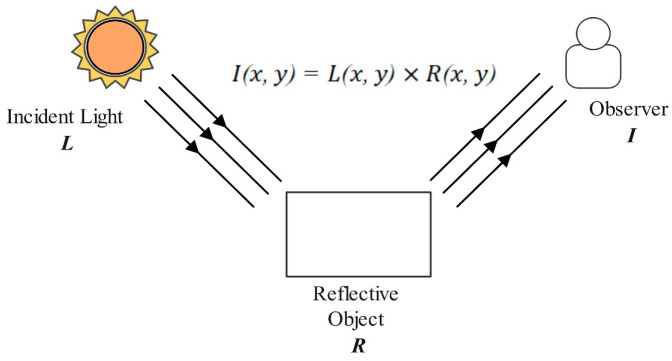
Retinex theory principle diagram.

The image partitioning based on attention mechanisms and Retinex theory is a composite process. It utilizes Retinex theory to simulate the human visual system’s perception of lighting and employs attention mechanisms to focus on key areas in the image. The principles of Retinex theory are illustrated in Figure 6.

Retinex theory posits that an observed image can be decomposed into an illumination component and a reflection component as follows:

(1) $$I\left(x,y\right)=L\left(x,y\right)\times R\left(x,y\right)$$

where $I\left(x,y\right)$ represents the observed image, x,y denote pixel positions in the image, $L\left(x,y\right)$ represents the illumination component, indicating the intensity and distribution of light in the scene, and $R\left(x,y\right)$ represents the reflection component, which reflects the inherent color and color characteristics of the object’s surface.

The purpose of employing an attention mechanism in this paper is to enable the model to focus on important parts of the image. In the context of image partitioning, this paper defines an attention weight $A\left(x,y\right)$, which is used to indicate the importance of each pixel. Therefore, the attention-weighted image is represented as follows:

(2) $$I_{A}\left(x,y\right)=A\left(x,y\right)\times I\left(x,y\right)$$

To further clarify how to apply different treatments to different areas, this paper defines a regional segmentation function $S\left(x,y\right)$ for the sample images. The regional segmentation function can divide the test image into several focused areas based on the image’s illumination component and attention fidelity. Specific treatments are then applied based on the characteristics of each region. For darker areas, such as the interior of a container ship’s hold, the method enhances the illumination component $L\left(x,y\right)$ to improve the visibility of image details. For high-light areas, brightness adjustment measures are taken to reduce halo effects. This focused strategy not only ensures the efficiency of the algorithm’s processing but also significantly reduces the required processing time. Through this method, the processing speed is enhanced while ensuring image processing quality, achieving rapid adaptation to complex image environments, and thus optimizing the balance between computational efficiency and effectiveness under the premise of ensuring image quality.

**Image Denoising Channel.** The image denoising channel aims to restore clear images from the haze effects caused by atmospheric scattering. When capturing images in hazy weather, tiny droplets or dust particles in the atmosphere scatter light, leading to a decline in image quality, which manifests as reduced contrast, color distortion, and blurred details. Additionally, images captured in such conditions often come with a higher noise level, so the image denoising process typically involves addressing blurring and noise issues while enhancing image details to improve visual quality.

In terms of image denoising, this paper initially uses a multi-scale wavelet decomposition algorithm to decompose low-quality images into low-frequency sub-images and multi-scale high-frequency sub-images. It then employs an adaptive Bayesian wavelet threshold estimation method to achieve nonlinear enhancement of different high-frequency sub-images, thus suppressing image noise information caused by environmental visibility and enhancing image details.

**Figure 7:**
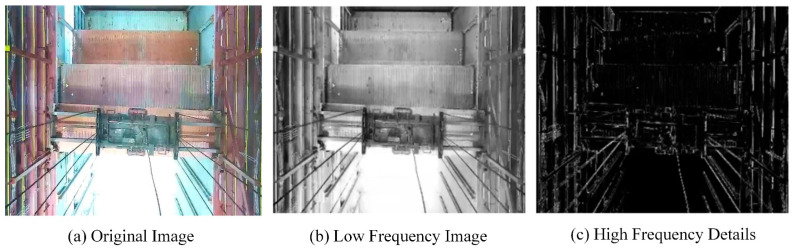
Sample images after wavelet decomposition. (**a**) Original image; (**b**) low frequency image; (**c**) high frequency image.

The multi-scale wavelet decomposition algorithm performs a hierarchical decomposition of images, allowing for the extraction of low-frequency components and multiple scales of high-frequency components of the image. The low-frequency components contain the main information of the image, such as the general contours and smooth areas, while the high-frequency components contain detailed information, such as edges and textures. The basic idea can be expressed by the following formula:

(3) $$I\left(x,y\right)=\sum_{s=1}^{S}\sum_{\omega \in \left\{LH,\;HL,HH\right\}}W_{s}^{\omega }\left(x,y\right)+L_{S}\left(x,y\right)$$

where $I\left(x,y\right)$ represents the original image and $W_{S}^{\omega }\left(x,y\right)$ represents the high-frequency wavelet coefficients at scale s, corresponding to the direction ω (horizontal details LH, vertical details HL, and diagonal details HH). $L_{S}\left(x,y\right)$ represents the low-frequency component at the final scale S, which is the approximate representation of the image. s is the scale or level of decomposition, and S is the maximum decomposition level. The samples collected in this paper are color images, therefore, wavelet decomposition is required for each color channel (typically the RGB channels). The processed channels are then recombined to form the complete image. The images after wavelet decomposition are shown in Figure 7.

During the initial decomposition process, this paper performs a first-level wavelet transform on the original image $I\left(x,y\right)$, decomposing $I\left(x,y\right)$ into a low-frequency component $L_{1}$ and high-frequency components $W_{1}^{LH}$, $W_{1}^{HL}$, $W_{1}^{HH}$. The low-frequency component reflects the general contours of the image, while the high-frequency components contain detailed information of the image. Subsequently, the low-frequency component is decomposed again, being further broken down into an even lower frequency component and new high-frequency components. This process is iterated until reaching the predetermined scale S. As an example of a second-level decomposition, the image is first decomposed into the first-level low-frequency component $L_{1}$ and high-frequency components $W_{1}^{LH}$, $W_{1}^{HL}$, $W_{1}^{HH}$. Then, $L_{1}$ is further decomposed into $L_{2}$ and high-frequency components $W_{2}^{LH}$, $W_{2}^{HL}$, $W_{2}^{HH}$. At this point, the multi-scale wavelet decomposition of the image can be represented as follows:

(4) $$I\left(x,y\right)=W_{1}^{LH}+W_{1}^{HL}+W_{1}^{HH}+W_{2}^{LH}+W_{2}^{HL}+W_{2}^{HH}+L_{2}$$

Multi-scale wavelet decomposition analyzes the frequency components of an image at different scales, capturing the image’s detail and structural information to achieve noise reduction. After decomposing low-quality images into low-frequency sub-images and multi-scale high-frequency sub-images, this paper utilizes an adaptive Bayesian wavelet threshold estimation method to achieve nonlinear enhancement of different high-frequency sub-images. This method applies an adaptive threshold based on Bayesian estimation to each high-frequency sub-image for nonlinear enhancement.

First, consider the representation of the image in the wavelet domain. For each high-frequency sub-image $W_{s}^{\omega }\left(x,y\right)$, where s represents the scale of wavelet decomposition and ω represents different directions, the set of wavelet coefficients is $c_{i,j}^{s}$. After obtaining the set of wavelet coefficients, it is necessary to determine the threshold. This paper uses the estimation of the noise level $\sigma_{n}$ to determine the threshold. The estimation of the noise level is accomplished by analyzing the variance of the wavelet coefficients in local regions of the image or other statistical methods.

After estimating the noise level, this paper determines the Bayesian threshold by minimizing Bayesian risk, with the following formula:

(5) $$\begin{array}{c} T=\mu \sigma_{n}\sqrt{2logN} \end{array}$$

(6) $$\begin{array}{c} \begin{array}{c} T_{s}^{\omega }=argmin_{T}E\left[L\left(c_{i,j}^{s},T\right)\right] \end{array} \end{array}$$

where, μ is an adjustable parameter, N is the number of data points, $L\left(c_{i,j}^{s},T\right)$ is the loss function, which quantifies the discrepancy between the true coefficients $c_{i,j}^{s}$ and the estimated coefficients under threshold T. $E\left[\cdot \right]$ represents the expectation operation, taking into account all possible noise and signal scenarios. This method utilizes the noise level to dynamically adjust the threshold, achieving effective denoising under various noise conditions.

Ultimately, for each wavelet coefficient $c_{i,j}^{s}$, the processing follows the following nonlinear logic:

(7) $$\begin{array}{c} {\hat{c}}_{i,j}^{s}=\left\{\begin{array}{ll} f\left(c_{i,j}^{s},T_{s}^{\omega }\right), & if\;\left|c_{i,j}^{s}\right|>T_{s}^{\omega }\\ 0, & otherwise \end{array}\right. \end{array}$$

where, $f\left(c_{i,j}^{s},T_{s}^{\omega }\right)$ represents a nonlinear function that adjusts the value of the coefficient $c_{i,j}^{s}$ based on its magnitude relative to the adaptive threshold $T_{s}^{\omega }$. The purpose of this function is to appropriately enhance an image while preserving image details. This nonlinear processing is based on whether the coefficients exceed the threshold to decide whether to retain the coefficient: coefficients exceeding the threshold are adjusted as they are considered to contain important image detail information, while those not exceeding the threshold are deemed to be noise and are set to zero.

The processed wavelet coefficients ${\hat{c}}_{i,j}^{s}$ are then used for image reconstruction via an inverse wavelet transform, achieving nonlinear enhancement of different high-frequency sub-images as follows:

(8) $$\begin{array}{c} \begin{array}{c} I^{\prime }\left(x,y\right)=InverseWaveletTransform\left({\hat{c}}_{i,j}^{s}\right) \end{array} \end{array}$$

**Figure 8:**
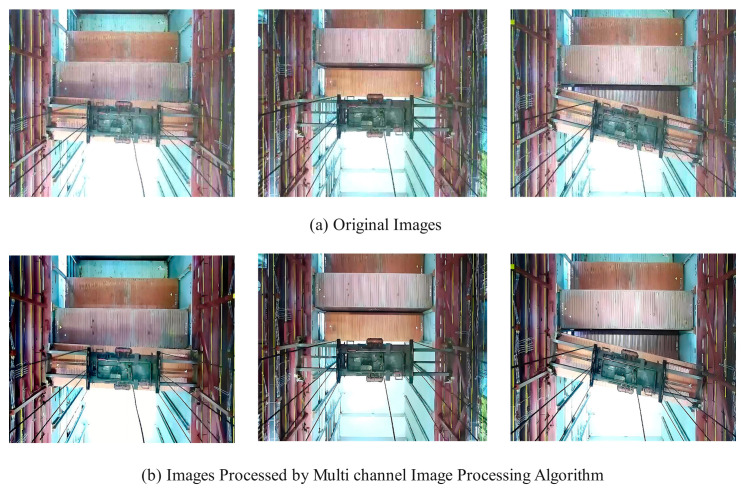
The preprocessed images. (**a**) shows the original images; (**b**) shows the images processed by the multi channel image preprocessing algorithm.

This process involves recombining the processed wavelet coefficients to form the enhanced image $I^{\prime }\left(x,y\right)$. This method, based on adaptive Bayesian wavelet threshold estimation, not only effectively enhances the high-frequency details of the image, thereby improving image clarity and visual quality, but also suppresses image noise to some extent. It is particularly suitable for cases where visual information loss is caused by environmental factors, such as haze. Its adaptive nature allows the threshold to dynamically adjust based on the characteristics of the image itself, thus enhancing image details while maintaining the naturalness and realism of the image. Examples of preprocessed images are shown in Figure 8.

#### 3.2.2. Object Detection Method Based on an Improved YOLOv5

Traditional object detection methods tend to fail in all-weather complex backgrounds such as docks, especially in cases of occlusion. Additionally, convolutional neural networks (CNNs) may include a large amount of redundant information when extracting object features, leading to incorrect object localization and a decrease in prediction accuracy. To address these issues, this paper proposes a method for detecting keypoints on spreaders based on YOLOv5, introducing a Mixed-Domain Attention Mechanism (MDAM). This method combines a Spatial Attention Mechanism (SAM) [24] and a Channel Attention Mechanism (CAM) [25] to enhance the model’s focus on important features, thereby improving detection performance in complex dock environments.

**Figure 9:**
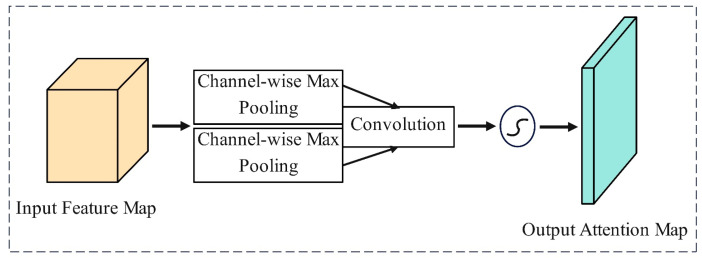
Spatial attention mechanism (SAM).

A SAM processes the input feature maps by performing channel-wise average pooling and max pooling, obtaining two spatial attention feature maps. These two attention maps are concatenated along the channel dimension to form a dual-channel feature map. Then, this map is convolved with a kernel, and a normalized attention map is obtained through an activation function. Finally, the attention map is element-wise multiplied with the original feature map to produce a weighted feature map, enabling the SAM to significantly enhance the model’s focus on important features, as shown in Figure 9.

**Figure 10:**
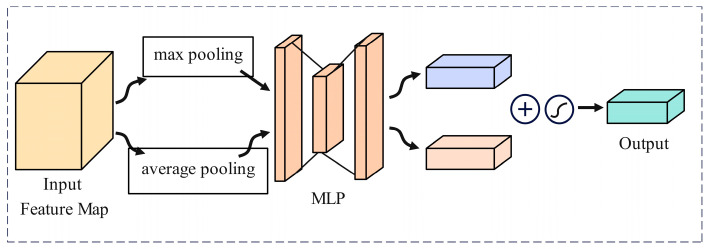
Channel attention mechanism (CAM).

A CAM obtains channel descriptors through global average pooling, and then generates channel weights through a series of fully connected layers. These weights are element-wise multiplied with the input feature map to enhance the representation of important channels, as shown in Figure 10.

**Figure 11:**
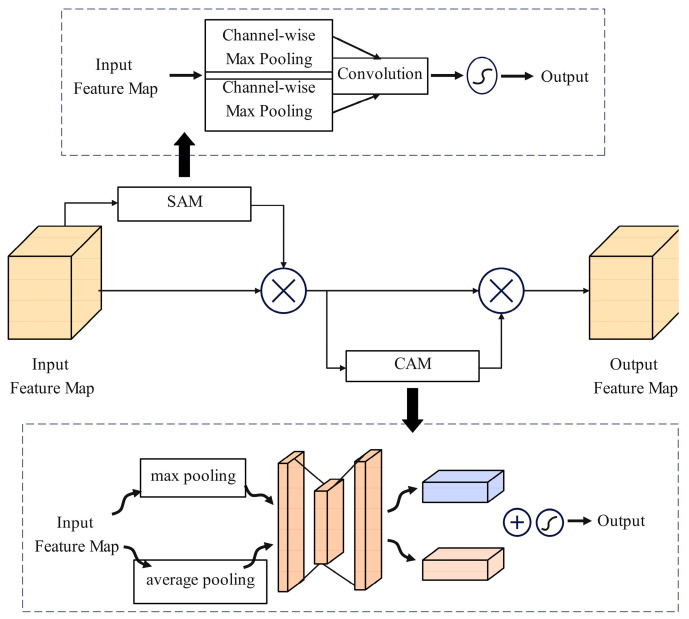
MDAD Module.

This paper combines the SAM and CAM modules sequentially into an MDAD module, as shown in Figure 11, with the specific steps as follows:

**Step One** Input a feature map F of size C×H×W. Channel average pooling and channel max pooling are used to compress the input features, generating feature layers of size 1×H×W each. These feature maps are then concatenated to form a dual-channel feature map of 2×H×W. Subsequently, a 7×7 convolution kernel is used to perform convolution operations to obtain $M\in R^{1\times H\times W}$, which is then passed through a Sigmoid activation function to produce a normalized attention map. The spatial attention map represents the importance of each positional information within the feature map.

**Step Two** Multiply the spatial attention map element-wise with the original feature map to obtain a weighted feature map ${F^{\prime }}_{out}=F\cdot M$.

**Step Three** Input the spatially weighted feature map into the CAM attention channel module. The input feature map ${F^{\prime }}_{out}$ undergoes global average pooling to generate channel weights $W_{c}\in R^{C}$. After normalizing the channel weights, the final weighted feature map is ${F^{\prime \prime }}_{out}={F^{\prime }}_{out}\cdot W_{c}$.

The MDAD module enhances the model’s sensitivity to important information by dynamically adjusting the weights of the feature maps during the feature extraction process. Specifically, the spatial attention mechanism identifies critical areas within the image, while the channel attention mechanism recognizes and emphasizes important channels in the feature maps. Combining these two attention mechanisms enhances feature expression across different dimensions, thereby improving detection accuracy and robustness.

**Figure 12:**
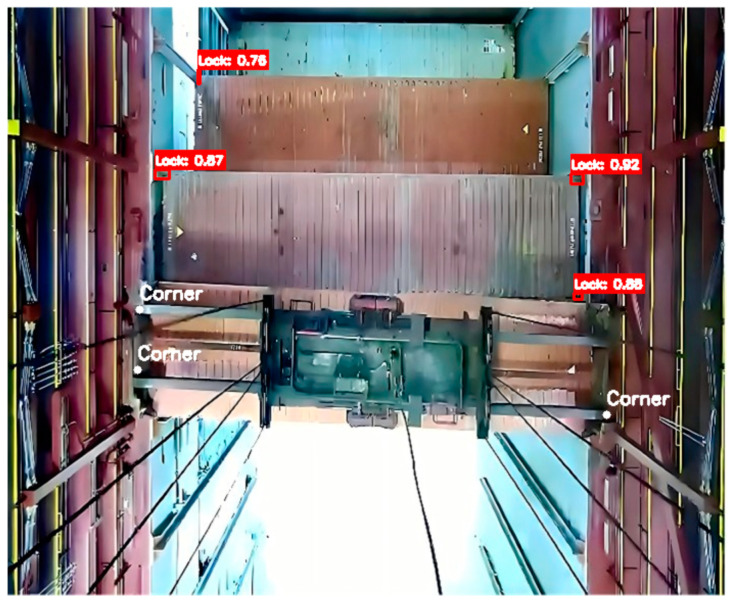
The detection results.

The detection results for the two-dimensional keypoints of the spreader obtained through the improved YOLOv5 network are illustrated in Figure 12.

The pixel coordinates of the four keypoints are obtained as $p_{i}=\left(u_{i},v_{i}\right)$, where i=1, 2, 3, 4.

#### 3.2.3. Spreader Three-Dimensional Position and Rotation Angle Measurement Model

The method proposed in this paper measures the rotation angle of the spreader in the camera coordinate system as well as the offset distance of the swinging spreader from the vertical position. During the lifting process of the spreader, the measurement system simultaneously detects the keypoints of the spreader and the lock holes of the containers in the ship’s hold. The coordinates and confidence level of the m-th detected lock hole are given as $lockhole_{m}=\left(u_{m},v_{m},confidence_{m}\right)$. When multiple lock holes are detected in the image, lock hole pairs are selected using the pixel coordinates values $\left(u_{m},v_{m}\right)$. The selection criterion is that the difference in the v coordinates between two lock holes should be within ±20 pixels as follows:

(9) $$\left|v_{m}-v_{n}\right|\leq 20$$

When multiple pairs of lock holes are detected in the image, for each pair that meets the criteria, calculate the average confidence level as follows:

(10) $$avg\_confidence=\frac{confidence_{m}+confidence_{n}}{2}$$

Select the lock hole pair with the highest avg_confidence to define the baseline for the spreader’s rotation angle. Therefore, the spreader’s rotation angle γ is calculated as follows:

(11) $$\gamma ={tan}^{-1}\left(\frac{v_{n}-v_{m}}{u_{n}-u_{m}}\right)-{tan}^{-1}\left(\frac{v_{2}-v_{2}}{u_{1}-u_{1}}\right)$$

**Figure 13:**
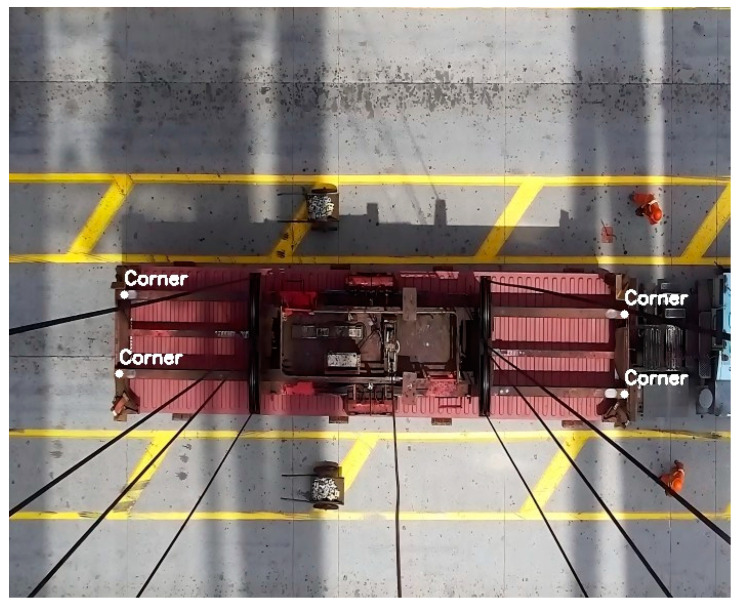
Preset Point.

The reference position for the spreader’s three-dimensional position is a preset point on the bracket, which is the coordinate point when the spreader descends vertically. The vertical distance d between the spreader and the camera is provided by the rope length sensor. The preset point is shown in Figure 13.

The pixel coordinates of the reference keypoints are $\left(u_{a},v_{a}\right)$, $\left(u_{b},v_{b}\right)$, $\left(u_{c},v_{c}\right)$, and $\left(u_{d},v_{d}\right)$, in sequence. Therefore, the changes in the spreader in the pixel coordinate system are as follows:

(12) $$\begin{array}{c} \Delta u=\frac{1}{4}\left(u_{1}+u_{2}+u_{3}+u_{4}-u_{a}-u_{b}-u_{c}-u_{d}\right) \end{array}$$

(13) $$\begin{array}{c} \Delta v=\frac{1}{4}\left(v_{1}+v_{2}+v_{3}+v_{4}-v_{a}-v_{b}-v_{c}-v_{d}\right) \end{array}$$

where Δu is the change in the spreader’s center along the u-axis in the pixel coordinate system, and Δv is the change in the spreader’s center along the v-axis. The camera focal length used in this paper is f. Since the camera is mounted on the trolley frame, the change in the vertical distance between the spreader and the camera can be obtained from the rope length sensor. At the reference position, the vertical distance between the spreader and the camera is D, and the vertical distance between the spreader and the camera is d. Therefore, the relationship between the displacement of the spreader in the pixel coordinate system and its displacement in the camera coordinate system is as follows:

(14) $$\begin{array}{c} \Delta x=f\frac{\Delta u}{d} \end{array}$$

(15) $$\begin{array}{c} \Delta y=f\frac{\Delta v}{d} \end{array}$$

(16) Δz=D−d

The final three-dimensional position of the spreader is $\left(\Delta x,\;\Delta y,\;\Delta z\right)$, and the spreader’s rotation angle is γ.

## 4. Experiment and Evaluation

### 4.1. Experimental Environment and Equipment Configuration

To validate the effectiveness of the proposed image-processing-based spreader pose measurement algorithm, a series of related experiments were conducted. The improved YOLOv5 was trained using a dataset annotated with spreader keypoints, and the experimental results were compared against target detection evaluation metrics.

The training environment parameters for this experiment are shown in Table 1 below.

**Figure 14:**
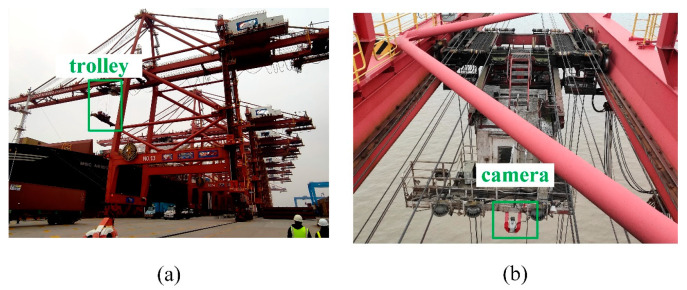
The actual installation of the camera. (**a**) Shows the red box indicates the quayside crane trolley; (**b**) shows the detail of the quayside crane trolley frame.

The camera used in this paper is a vertically mounted camera with a pixel resolution of 1920 × 1080 and an fps of 24. The actual installation of the camera is shown in Figure 14. In Figure 14, Figure 14a shows the red box indicating the quayside crane trolley, and Figure 14b shows the details of the quayside crane trolley frame with the green box indicating the actual installation position of the camera.

**Figure 15:**
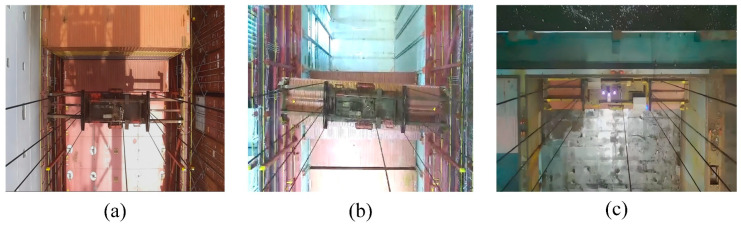
(**a**) Shows an image sample under daytime lighting conditions; (**b**) shows an image sample under nighttime lighting conditions; and (**c**) shows an image sample under rainy weather conditions.

The dataset collected a total of 5670 images, which were divided into training and testing sets at a ratio of 8:2. This dataset includes samples from various lighting conditions such as daytime, nighttime, and rainy weather, as specifically shown in Figure 15.

The performance of the quayside crane spreader pose measurement system designed in this paper mainly depends on the following aspects: the detection accuracy of the spreader keypoints and lock holes, the real-time performance of pose measurement, and the accuracy of pose measurement. Therefore, the experimental part focused on three core evaluation metrics: model measurement accuracy, model inference speed, and the single operation time of the spreader on the container. By comparative experiments, this paper evaluated the system’s performance on these key indicators in detail to validate the effectiveness and practicality of the proposed system.

### 4.2. Model Estimation Accuracy Experiment

To test the effectiveness of the image preprocessing algorithm and the improved YOLOv5 algorithm for detecting the spreader keypoints and the lock holes on the container’s upper surface, a comparative experiment was conducted using the original YOLOv5 algorithm and the improved YOLOv5 algorithm.

The evaluation metrics used in the experiment include the algorithm’s Precision, Recall, and Mean Average Precision (mAP).

Precision is the proportion of positive identifications (i.e., detected targets) that are correct. It is expressed by the following formula:

(17) $$Precision=\frac{TP}{TP+FP}$$

where TP represents the number of true positives, and FP represents the number of false positives.

Recall is the proportion of actual positives that are correctly identified by the model. It is expressed by the following formula:

(18) $$Recall=\frac{TP}{TP+FN}$$

where FN represents the number of instances that are actual positives but are incorrectly predicted as negatives.

mAP is the average of the Average Precision (AP) for each category. This study primarily utilizes two metrics: mAP@0.5 and mAP@0.5:0.95, to more comprehensively evaluate the performance of object detection models. mAP@0.5 refers to the mAP value when the IoU threshold is set at 0.5, meaning that a detection is considered valid only if the predicted bounding box has an IoU of at least 0.5 with the true bounding box. mAP@0.5:0.95, on the other hand, is the mAP calculated over an IoU threshold range from 0.5 to 0.95.

**Figure 16:**
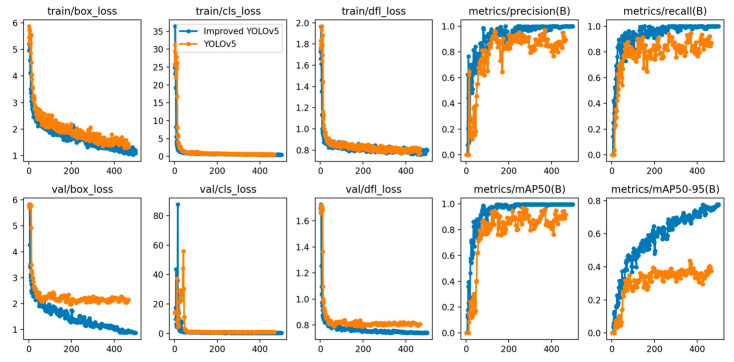
The training results of the improved YOLOv5 algorithm and YOLOv5 algorithm.

The training results of the improved YOLOv5 network compared to the original YOLOv5 network are shown in Figure 16, where the blue line represents the improved YOLOv5 network and the orangeline represents the original YOLOv5 network. The horizontal axis in Figure 16 represents the number of epochs during the training process.

As shown in Figure 16, when comparing the loss functions of the two algorithms, the improved YOLOv5 surpasses the original YOLOv5 in the speed of reducing bounding box regression loss and reaches convergence faster, with a final bounding box regression loss of 0.47. In terms of Precision, when the epoch count is between 0 and 200, the precision curves of both algorithms exhibit oscillations with similar growth rates. However, after surpassing 200 epochs, the improved YOLOv5 gradually begins to converge and stabilizes first. In terms of mAP comparison, the improved YOLOv5’s mAP@0.5 stabilizes after 250 epochs, while the original YOLOv5 still shows fluctuations. Furthermore, the improved YOLOv5 consistently outperforms the original algorithm on the mAP@0.5:0.95 metric, especially around 150 epochs of training, where its performance is significantly better than the original algorithm. This indicates a noticeable improvement in the accuracy of target identification and localization, as well as overall algorithm performance in the improved YOLOv5.

The above analysis demonstrates how the improvement module enhances network performance through the trend of the curves. Next, the ablation experiment in Table 2 will detail the specific impact of this improvement module on four key metrics: Precision, Recall, mAP@0.5, and mAP@0.5:0.95.

The improved YOLOv5-based algorithm for detecting spreader keypoints and container lock holes shows enhancements in precision (P), recall (R), and mean precision (mAP@0.5), and mAP@0.5:0.95. After only adding the image preprocessing algorithm, compared to the original YOLOv5, the improved algorithm shows increases of 0.8% in Precision, 7.3% in Recall, 2.6% in mAP@0.5, and 3.8% in mAP@0.5:0.95. After adding the attention module, the improvements in these metrics compared to the original model are 0.6%, 7%, 3.6%, and 5.3%, respectively. When both the image preprocessing algorithm and attention module are integrated, the enhancements in these metrics are even more significant compared to the original YOLOv5 model, at 7%, 10.4%, 8.3%, and 17.6%, respectively. These results effectively validate the efficacy and higher recognition accuracy of the proposed spreader keypoints and container lock hole detection algorithm.

**Figure 17:**
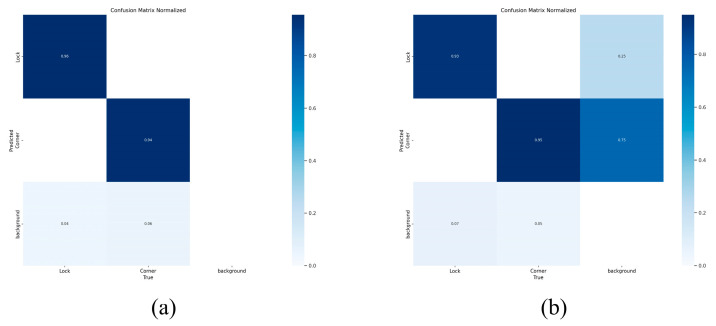
Confusion matrix. (**a**) Shows the confusion matrix for the improved YOLOv5; (**b**) shows the confusion matrix for the original YOLOv5.

Figure 17 displays a confusion matrix. The parameters on the diagonal of the matrix represent the recall rate for each class of object, and the level of recall directly reflects the accuracy of classification. Figure 17a shows the confusion matrix for the improved YOLOv5, while Figure 17b shows the confusion matrix for the original YOLOv5. It is evident from the figures that the improved YOLOv5 algorithm has significantly enhanced accuracy in sample classification and superior detection performance.

**Figure 18:**
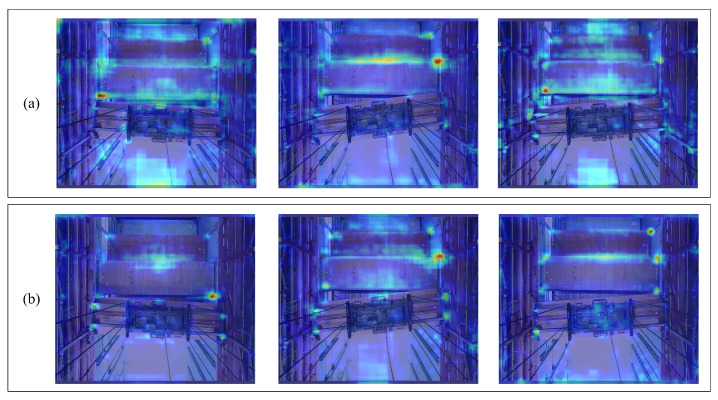
Grad-CAM visualization analysis. (**a**) Shows the Grad-CAM visualization results for the original YOLOv5; (**b**) shows the Grad-CAM visualization results for the improved YOLOv5.

This paper further conducted a Grad-CAM visualization analysis of both the improved YOLOv5 network and the original YOLOv5 network. The visualization results are shown in Figure 18, where Figure 18a shows the Grad-CAM visualization results for the original YOLOv5, and Figure 18b shows the Grad-CAM visualization results for the improved YOLOv5.

As shown in Figure 18, it is evident that the original YOLOv5 algorithm has poorer capability in extracting effective features, is easily disturbed by redundant information in images, and tends to focus on more scattered areas. In contrast, the heatmaps of the improved YOLOv5 model show that the darker areas are mainly concentrated around the lock holes and keypoints of the spreader, indicating that the features extracted by the improved model align with the expected features. This demonstrates that the improvement methods proposed in this paper effectively aid in extracting key features and significantly reduce the interference from irrelevant features.

### 4.3. Engineering Application Comparative Experiment

**Figure 19:**
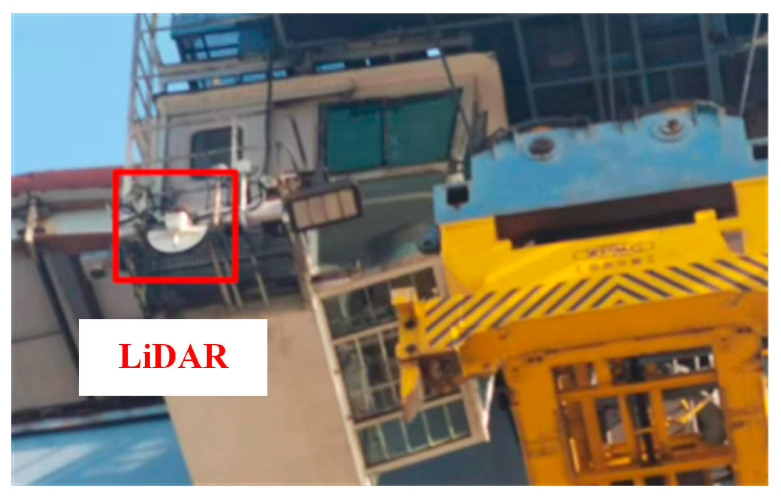
The installation of LiDAR equipment.

Currently, the three-dimensional positioning and attitude measurement of port container spreaders primarily utilize LiDAR-based technologies. The installation of LiDAR equipment used on the engineering site is shown in Figure 19.

To verify the effectiveness of the machine vision-based measurement method proposed in this paper in practical applications, 100 operational cycles recorded on video were analyzed to calculate the average duration of a complete loading and unloading process. The average time for a single cycle of measuring container pose using LiDAR and automatically picking up the container was 124.71 s. The comparison of field test data is shown in Table 3.

**Figure 20:**
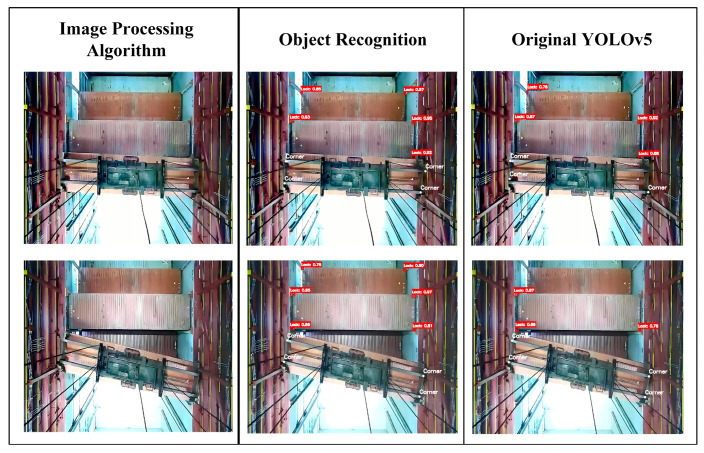
The recognition results.

Additionally, using the proposed detection method for automated operations, the average operation time for 100 datasets was 96.34 s: an improvement of 28.37 s. The recognition results are shown in Figure 20.

## 5. Conclusions

The accurate measurement of the 3D positioning and posture of container spreaders is vital for the safe and efficient transfer of containers in automated shore-based container cranes. This study introduces a method utilizing a single fixed-focus vertical camera for high-precision measurement of the spreader’s 3D position and rotation angles. By employing an image preprocessing technique and integrating an improved YOLOv5 network with an attention mechanism, we significantly enhanced the detection accuracy of spreader keypoints and container lock holes.

Compared to traditional methods, the proposed single-camera-based approach demonstrated superior accuracy. The improved algorithm showed marked improvements in precision, recall, and mean precision, validating its effectiveness for detecting spreader keypoints and container lock holes. Additionally, the proposed detection method reduced operation times, confirming its practical applicability and efficiency in enhancing the automation of shore-based container cranes.

## Figures and Tables

**Table 1:** The training environment parameters for this experiment.

Configuration	Name	Parameter Settings
Hardware Environment	GPU	NVIDIA GeForce RTX 3090
	CPU	Intel Xeon Processor E52680 v4
Software Environment	Operating System	Ubuntu 20.04
	Programming Language	Python = 3.8
	Machine Learning Library	Pytorch = 1.8

**Table 2:** Ablation experiment results of YOLOv5 with Image Preprocessing and Attention Module.

Methods			P (%)	R (%)	mAP @0.5 (%)	mAP @0.5:0.95 (%)
YOLOv5	Image Preprocessing	Attention Module				
√	×	×	91.6%	86.3%	90.0%	76.4%
√	√	×	92.4%	93.6%	92.6%	80.2%
√	×	√	92.2%	93.3%	93.6%	81.7%
√	√	√	98.6%	96.7%	98.3%	94.0%

**Table 3:** The comparison of field test data.

Methods	Speed (FPS)	Operation Time (Seconds)
YOLOv5	10.23	/
Lidar	7.87	124.71
Ours	13.76	96.34

## Data Availability

The data used in this study did not involve any public datasets.
